# Supplementary material for: Comparative Study of the Nutritional Composition and Antioxidant Ability of Soups Made from Wild and Farmed Snakehead Fish (Channa Argus)
Source: Foods. 2022 Oct 21;11(20):3294. doi: 10.3390/foods11203294 (PMC9601314; doi:10.3390/foods11203294)
Supplement: Supplementary file 1 [file foods-11-03294-s001.zip › foods-1940732-supplementary.pdf]

Supplementary Materials:

Table S1. Food nutritional composition and growth environment of farmed and wild snakehead fish

Food	Farmed		Wild					
	Feed [1-3]		Trash fish [4-7]		River prawns [8,9]		Frog [10,11]	
	Wet base	Dry base	Wet base	Dry base	Wet base	Dry base	Wet base	Dry base
Moisture	10.0~12.5		69.5~75.88		71.0~81.3		78.14~85.87	
Crude protein	40.0~45.0	44.44~51.43	16.09~19.55	58.62~64.10	15.18~20.8	66.0~71.72	10.81~19.07	76.50~87.24
Crude fat	5.0~6.0	5.56~6.86	2.59~4.51	10.74~16.43	1.9~2.68	10.16~11.37	0.17~0.90	0.92~6.37
Fiber	5.0~8.0	5.56~9.14	-	-	-	-	-	-
Crude ash	14.0~18.0	15.56~20.57	0.91~3.85	3.32~15.96	1.5~4.0	5.17~21.29	0.52~1.1	3.68~5.52
Calcium	0.5~5.0	0.56~5.71	0.29~1.10	0.95~4.48	0.23~1.04	0.80~4.41	0.0107~0.49	0.054~3.47
Phosphorus	0.5~5.0	0.56~5.71	0.57~0.69	1.87~2.82	0.047~0.76	0.16~3.22	0.0064~0.91	0.032~4.82
Amino acid	2.1~2.3	2.33~2.63	-	-	-	-	-	-
Habitual density	3000~6000 tails/30m2 1		<10 tails					
Illumination	High light intensity		Low light intensity					
Water temperature	The temperature is stable with slight change		Great temperature change					
Way of eating	Feed on time		Hunting					

References:

1. Feed A consists of imported fish meal, starch, soybean meal, fish oil, calcium dihydrogen phosphate, vitamins, multivitamins and organic trace elements. The ingredients refer to the nutrient composition table of snakehead feed, which comes from Zhejiang Dongyu Biotechnology Co., Ltd.;
2. Feed B consists of imported high-quality fish meal, flour, soybean meal, yeast powder, organic chelated minerals, multiple vitamins, trace elements, etc. The ingredients refer to the nutrient composition table of snakehead feed, which comes from Jiangmen Hengsheng Industrial Co., Ltd.
3. Zhejiang Lianxing Feed Technology Co., Ltd. An extruded compound feed for blackfish: CN201510830228.8.2016-03-30.
4. Wang, Y.H.; Ding, W.; Chen, J.; Wang, H. C.; Luo, G. L.; Xing, J.; Xin, J. Q. Effects of feeding compound feed and chilled trash fish on the growth rate and feed cost of turbot. *Jiangsu Agricultural Science* **2016**. 44(07):282-285.
5. Li, J. Q.; Lin, J. B.; Zhu, Q. G.; Li, C. L. A comparative experiment on the effect of artificial compound feed and small trash fish on raising red-spotted grouper. *Taiwan Strait* **2004** (02): 167-173.
6. Huang, Z. C., Chen, D. H., Lin, J. B., Lin, K. B., Zhu, Q. G., Liang, P., Zheng, L. Y., and Qiu, F. Y. Effects of compound feed and small trash fish on the growth performance of grouper oblique. *Feed Research* **2012**(09): 1-4.
7. Chen, D. H.; Zheng, L. Y.; Lin, J. B.; Zhu, Q. G.; Liang, P.; Lin, K. B.; Huang, Z. C.; Qiu, M. L. Study on the effects of different feeds and small trash fish on the growth and immunity of grouper serrata. *Journal of Fujian Agriculture* **2013**. 28(04):309-314.
8. Cui, J.; Meng, C.; Liu, B. L.; Li, W. D. Nutrient content and evaluation of five main species of freshwater shrimp in Anhui Province. *Health Research* **2020**. 49(06): 962-968.
9. Zhuang, P.; Song, C.; Zhang, L. Z. Nutrient composition comparison between white shrimp and Macrobrachium japonicus in the Yangtze Estuary. *Journal of Zoology* **2008**(04):822-829.
10. He, X. R.; Wang, X. Q.; Fan, W. J.; Zhou, X. W. Biological characteristics and nutritional composition analysis of Sangzhi toe ditch frog. *Journal of Hunan Agricultural University (Natural Science Edition)* **2008**(04):482-484.
11. Ouyang, F.; Chen, Z.; Shi, L.; Chen, X. H. Analysis of the nutritional components of the anal frog of Taihang longa. *Journal of Tianjin Normal University (Natural Science Edition)* **2015**. 35(03):128-129+136.

Figure S1

Aspect ratio：4.74±0.16b

BMI：5.20±0.26a

Farmed snakehead

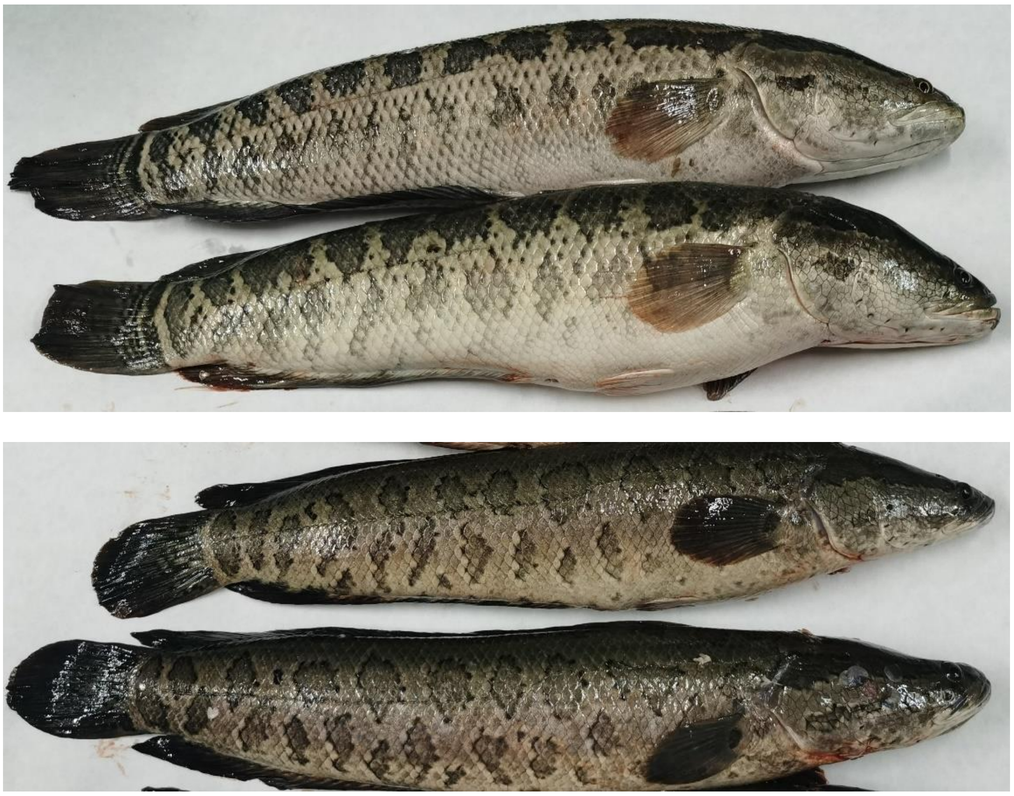

Wild snakehead

Aspect ratio：5.81±0.04a

BMI：4.24±0.11b

Figure S1. Appearance and morphological parameters of farmed and wild snakehead fish. Different lowercase letters indicate significant difference in the body mass index between the farmed and wild snakehead fish ($P < 0.05$)

foods MDPI

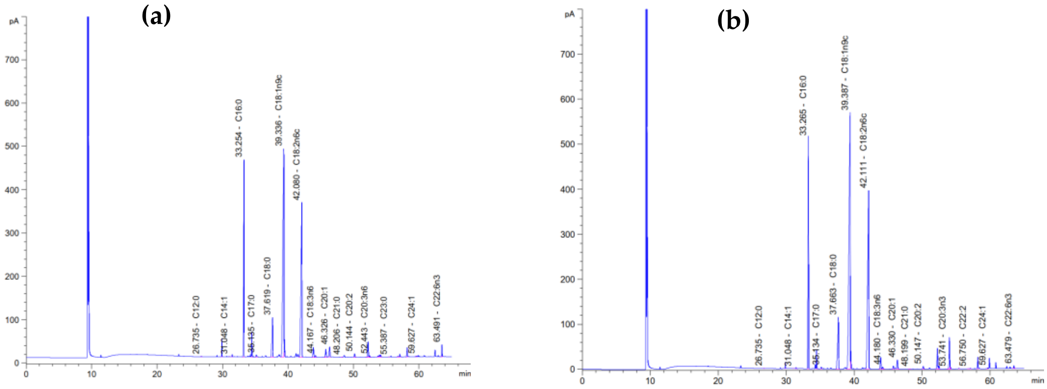

Figure S2. The GC-MS chromatogram of fatty acid analysis. a: farmed snakehead fish soup; b: wild snakehead fish soup.
